# The Chronic Stages of Bovine *Fasciola hepatica* Are Dominated by CD4 T-Cell Exhaustion

**DOI:** 10.3389/fimmu.2017.01002

**Published:** 2017-08-21

**Authors:** Divya Sachdev, Kevin C. Gough, Robin J. Flynn

**Affiliations:** ^1^School of Veterinary Medicine and Science, University of Nottingham, Loughborough, United Kingdom; ^2^Department of Infection Biology, Institute of Infection and Global Health, University of Liverpool, Liverpool, United Kingdom

**Keywords:** *Fasciola hepatica*, chronic helminth infection, T-cell responses, IL-10, IL-2, hyporesponsive, anergy, Foxp3

## Abstract

*Fasciola hepatica* infection of ruminants leads to non-resolving chronic infection, as patency develops, there is switching to a TGF-β and IL-10 led response. Here, we explore the responses of CD4 T-cells within the major draining lymph nodes. We found minimal expression of Foxp3 within CD4 cells but elevated levels within the γδ (WC1^+^) population. There is a strong T-cell-intrinsic exhaustion phenotype within the hepatic lymph node (HLN) characterized by a lack of antigen-specific proliferation and cytokine secretion. CD4 T-cells recovered from the HLN had high levels of PD-1 expression and low levels of IL-2 secretion. Exogenous IL-2 partially rescued this defect; when combined with neutralization of IL-10 and TGF-β, full restoration of proliferation, and cytokine production was achieved. Moreover, there is a clear uncoupling of the mechanisms that facilitate this regulation with parasite-specific proliferation and cytokine secretion being governed by independent means. These data would suggest that there is a CD4 T-cell-intrinsic regulation in place early in chronic infection, potentially leading to failure in resistance to reinfection.

## Introduction

*Fasciola hepatica* is known to establish chronic infections in its bovine host despite the development of a strong type-2/CD4^+^ Th2 response (1). As such, animals fail to develop resistance to reinfection as has been demonstrated experimentally (2). Reasons underlying this have been suggested to be similar to those seen in other models of helminth infection. *Schistosoma mansoni* infection shows a strong increase in the number of CD4 Foxp3^+^ Treg cells in parallel with the Th2 response (3); however, a later study using live infection, as opposed to schistosome egg antigen challenge (3), suggested that the control of the response was not dependent on Foxp3^+^ T-cells but on a population of CD4^+^CD25^−^CTLA4^+^ IL-10-secreting cells (4). Further studies using *Trichinella spiralis* showed that regulation of the response against the muscle resident phase relied not only upon Foxp3^+^ CD4 T-cells but also upon IL-10 from a distinct cellular source (5); echoing those findings of Helmby and Grencis (6) who had demonstrated that IL-10 was effective at restraining the cellular response directed at larvae but not intestinal adults. These findings would suggest that Foxp3 expression alone is not responsible for the regulatory response to tissue helminths.

Previous studies of *F. hepatica* in the murine model suggest that CD4 Foxp3^+^ T-cells expand but these cells make up one of two major IL-10^+^ T-cell populations, the second being most likely Tr1 (CTLA4^+^CD25^+^) cells. However, while this was the case in the peritoneal cavity, no expansion of Foxp3^+^ cells were seen in the mesenteric lymph node (MLN) implicating the Tr1 population in IL-10 production and regulatory functions (4). More recently, a murine study has definitively classified the CD4 T-cell population as anergic; anergy could be induced by both live infection and injection of a tegumental antigen preparation (FHTEG). Aldridge and O’Neill reported that PD-1, CTLA4, and GRAIL (RNF128) were all upregulated in the splenic CD4 population after antigen injection (7). The effect of FHTEG was found to be multimodal *via* both DCs and their expression of the mannose receptor or directly on the CD4 population itself; reflecting the heterogeneous nature of FHTEG. The loss of antigen-specific responses, both proliferation and cytokine production, could be restored by the addition of IL-2. In the bovine model of infection, we have previously shown at a peripheral level, that both TGF-β and IL-10 played a role in limiting IL-4 and IFN-γ production. The levels of IL-10 and TGF-β increased as infection progressed while proliferation capacity continued to diminish in the face of an expanding lymphocyte number (8). There appears to be multiple mechanisms responsible for the suppression of CD4 T-cell parasite-specific responses that largely exclude Foxp3; should these all arise from a single cell population effectively targeting them *via* adjuvant to overcome their effects will be simpler than if multiple cell populations act in concert to suppress the CD4 response.

The mechanisms by which the adaptive immune system is restrained is of foremost importance for any future vaccine prospects; as pre-existing anergic antigenic-specific responses could compromise the potential of any vaccine under field conditions. In particular, this is important to address within the liver environment where ongoing immune responses need to be controlled carefully to balance any potential pathology with benefits as a result of parasite clearance. This raises several questions for the regulation of the *F. hepatica* CD4 T-cell response; herein, we examined the hepatic lymph node (HLN) CD4 T-cell pool for functional capacity during chronic patent infection with *F. hepatica*.

## Materials and Methods

### Experimental Infection

Male Holstein × Friesian cattle at 3 months of age were used, animals were tested for prior exposure to *F. hepatica* before experimental infections using both serological analysis and fecal egg counts (FECs). Animals were infected with 250 metacercariae from Ridgeway Scientific and maintained until 13 weeks of postinfection. Throughout the experiment, animals had *ad libitum* access to feed and water. After, the infection period animals were euthanized by stunning with a captive bolt and exsanguination. Blood was collected weekly and, at PM, lymph nodes were removed and collected into sterile PBS.

Parasite-specific LFH IgG1 was determined as previously described using LFH (10 µg/mL) as a capture antigen (9), plates were probed with serially diluted serum samples. Thereafter, bound IgG1 was detected using mouse-anti-bovine IgG1:HRP (Abdserotec clone IL-A60) and TMB. Additionally, entire livers were removed and examined to determine the final parasite burden, parasites were determined to be mature or immature on the basis of body shape and the development of the “shoulder” area of the fluke as previously described (10, 11). FECs were performed on 1 g of feces using the standard sedimentation technique.

### Ethics Statement

Experiments were conducted under Home Office License PPL 30/3239 in accordance with Animal Scientific Procedures Act 1986 and ethical approval for the study was provided by the University of Nottingham SVMS and ADAS UK Animal Welfare and Ethics Review Committee. Animal involved in experiments had their suffering minimized and their welfare was as outlined in the guidance provided by EU Directive 2010/63/EU.

### Tissue Culture

Single cell suspensions were prepared from the lymph nodes by forcing through a 70 µm cell strainer, thereafter, red blood cells were lysed with ammonium chloride lysis buffer. Cells were washed twice in 1× PBS and resuspended at 10^7^ cells/mL for cell separation. Miltenyi magnetic cell separation was used, as per the manufacturer’s instructions, following a primary incubation with the mouse anti-bovine CD4 monoclonal antibody (AbDserotec—clone CC30; mouse IgG1) at 1/50 dilution followed by incubation with secondary Miltenyi magnetic beads coated with anti-mouse IgG as per manufacturer’s instructions. Purified cells were cultured at a final density of 2 × 10^6^/mL and stimulated with endotoxin-free total adult antigen extract (LFH) at 10 µg/mL or anti-bovine CD3 (VMRD—clone MM1A) that had been coated onto tissue culture plates at 1 µg/mL overnight. Where indicated, anti-IL-10 (AbDserotec—clone CC320) (12, 13) or anti-TGF (R&D Systems—polyclonal AB-100-NA) (14) were used at 1 µg/mL, alternatively cells were cultured in the presence of recombinant bovine IL-2 (Kingfisher Biotech RP0026B at the indicated concentrations), all cultures were in RMPI-1640 with 10% heat-inactivated fetal calf serum (FCS), penicillin (100 U/mL), and streptomycin (100 µg/mL).

For coculture, experiments requiring PBMCs cells were purified as before (8); PBMCs were subsequently stored in liquid nitrogen in 80% FCS/20% DMSO. After thawing, cells were washed immediately twice in complete RMPI prior to separation of CD4 T-cells as above. Cells were then cocultured together in the indicated ratios prior to analysis for either IFN-γ or proliferation using the MTT assay (Sigma-Aldrich).

### FACs Analysis

Single cell suspensions were stained with combinations of antibodies indicated below for 25 min at 4°C protected from the dark. Cells were acquired on a BD FACS Aria (BD Biosciences, San Jose, CA, USA) and gated on forward and side scatter to exclude damaged cells and debris. Cells were stained with combinations of the following; anti-bovine CD4 (Alexa Fluor^®^ 647 clone CC8, mouse IgG2a, AbDserotec), anti-bovine CD25 (RPE clone ILA111, mouse IgG AbDserotec), anti-bovine WC1 (FITC clone CC15, AbDserotec), anti-bovine Foxp3 (APC FJK-16s, rat IgG2a, eBioscience), anti-bovine CD62L (FITC clone CC32 AbDserotec), and anti-bovine CD45RO (RPE clone IL-A116, AbDserotec). Appropriate isotype and unstained controls were captured. Data were analyzed using FlowJo version 7.6.1 analysis software (TreeStar, San Carlos, CA, USA).

### Cytokine Measurements

Supernatants were tested for the presence of cytokines using commercial ELISAs as follows; TGF-β (Promega-G7591), IL-4 (Thermofisher-ESS0031), IFN-γ (MABtech-3119-1H-6), IL-5 (LSBio LS-F6133), IL-13 (Kingfisher Biotech VS0148B), and IL-2 (Kingfisher Biotech DIY1100B). For the detection of IL-10, IL-10 capture antibody (AbDserotec, clone CC318) was used at 6 µg/mL and detection IL-10 antibody (AbDserotec, biotin-conjugated clone CC320) was used at 2 µg/mL as previously described (15), with the inclusion of recombinant IL-10 as a standard curve. All assays were carried out as per manufacturer’s instructions with optical densities captured on a LT-4000 microplate reader (Labtech, UK) with standard curves used for quantification throughout.

### qPCR

To measure PD-L1 and CTLA4 *via* qPCR, RNA was isolated from freshly prepared cells and converted to cDNA using the GoScript Reverse Transcription Kit (Promega). Primers for *pdcd1* and *ctla4* (Applied Biosystems #4351372 and #4351372) and conditions used are as previously reported (16). Assays were run in quadruplicate on a Roche Lightcycler II, data were analyzed using the ΔΔCT method to determine fold expression changes relative to rpl19 (Applied Biosystems #4331182), which has previously been shown to be a suitable housekeeping gene for immunological analysis (17).

### Statistical Analysis

Data were plotted within GraphPad Prism (La Jolla, CA, USA), a *P-*value cutoff of 0.05 was taken as significant, and details of specific tests used are given in the relevant figure legends.

## Results

### Cytokine Responses of CD4 T-Cells at Draining Lymph Nodes of *F. hepatica* Infected Animals

**Figure 1:**
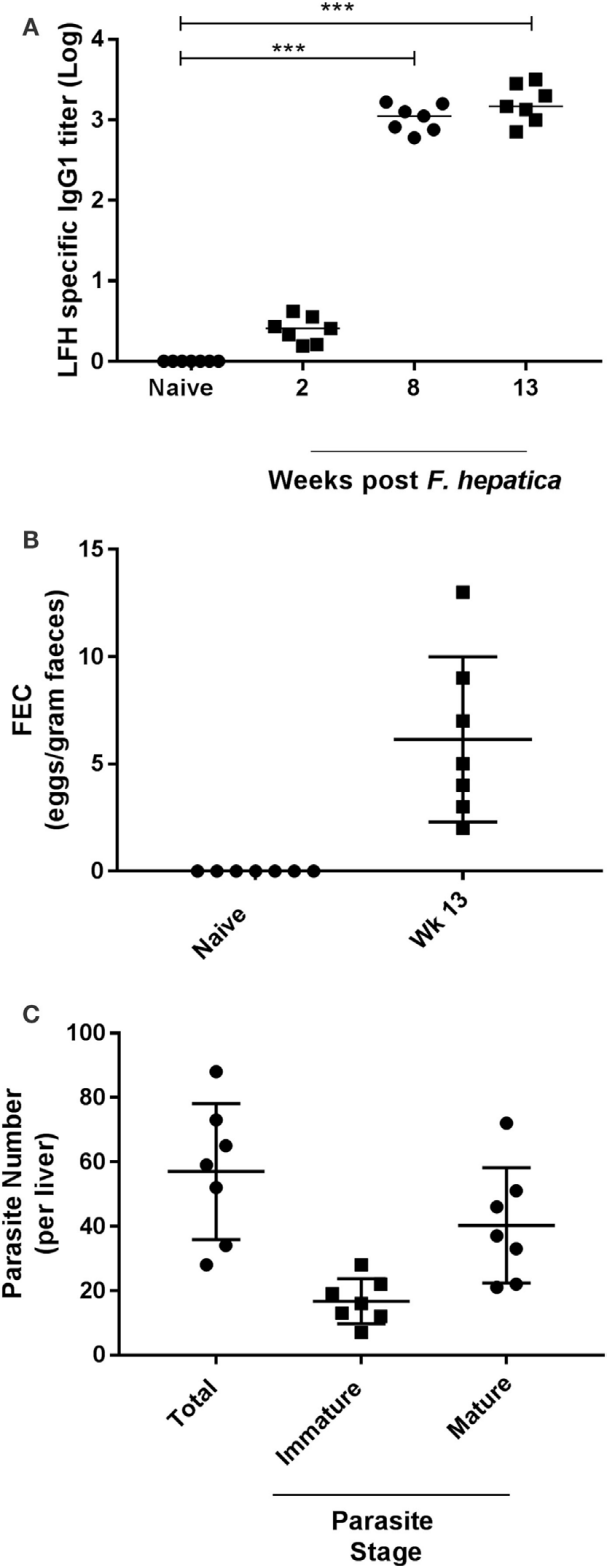
Parasitological parameters and seroconversion of infected animals. **(A)** Animals were examined for pre- and postinfection parasite-specific IgG1 antibody titers. Using a capture ELISA endpoint, log antibody titers were determined for each animal at the indicated time points. **(B)** Fecal egg counts (FEC) were performed using 1 g of feces from each animal before (naïve) and after infection at week 13. Data are presented as eggs per gram of feces. **(C)** At postmortem, livers were removed and examined from each animal. Total parasite numbers were determined for each liver. Additionally, parasites were classified as being immature or mature for each animal also. Each data point presented represents and individual animal with medias for each time point shown on the graph; data in **(A)** were examined for significance using a Kruskal–Wallis with Dunn’s multiple comparisons where ***P < 0.001.

Preinfection examination of the animals revealed neither specific anti-*Fasciola* IgG1 antibodies (Figure 1A) nor parasite eggs present within feces (Figure 1B). However, as infection progressed, the development of parasite-specific IgG1 became clear at week 2 postinfection with titers reaching a plateau by week 8 and remaining at these levels until completion of the trial (Figure 1A). Similarly, when animals were examined for the presence of parasite eggs in the feces, we found all animals to be egg positive, with a range of 2–13 eggs/g and a median count of 5 (Figure 1B). At postmortem, all livers were examined for the presence of infiltrating parasites in the bile ducts, gall bladder, and throughout the tissues; total parasite numbers and both immature and mature forms were counted (Figure 1C). All animals harbored parasites in their liver at examination; the range of total parasites recovered was 28–88, while all animals had greater numbers of mature parasites (range: 21–72) compared to immature parasites (range: 7–28; Figure 1C).

**Figure 2:**
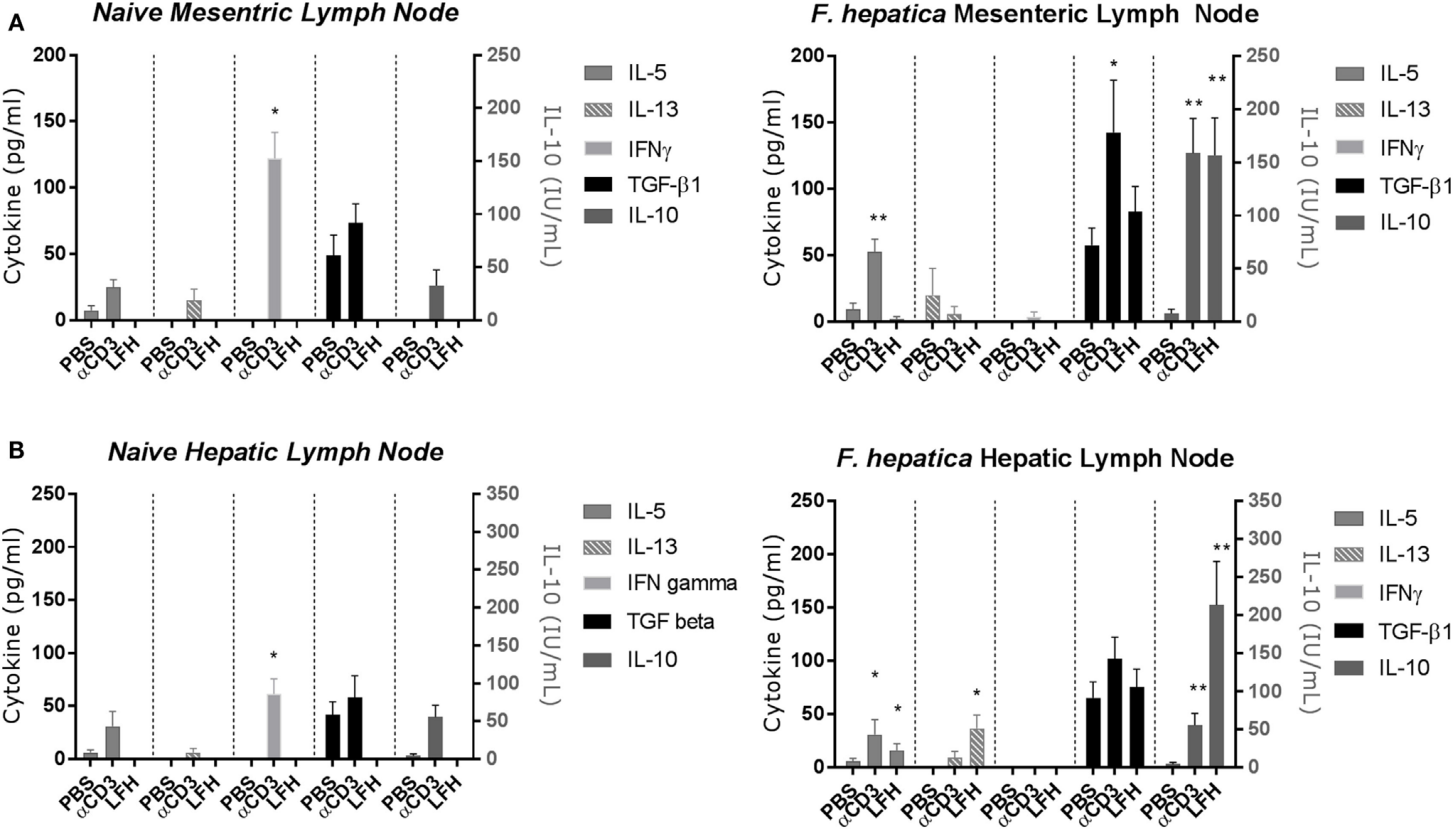
Cytokine responses of the CD4 T-cell compartment within draining lymph nodes of naïve animals (left panels) or those undergoing an experimental chronic *Fasciola hepatica* infection (right panels). At 13 weeks of postinfection, CD4 T-cells were isolated from the mesenteric lymph node (MLN) **(A)** and hepatic lymph node (HLN) **(B)** and cultured with anti-CD3 (αCD3) or LFH. Thereafter, cytokines were measured in cell-free supernatant by ELISA. There was significant production of IL-10 in both compartments with both treatments, TGF-β was produced above basal levels with stimulation with αCD3 in MLN, IL-5 was produced with αCD3 treatment in both compartments and with LFH in HLN only. LFH stimulated the production of IL-13 in HLN. No other combinations of treatment and lymph node produced significant levels of cytokine. Note the different units for IL-10. Graphs represent mean ± SEM of seven animals, with technical triplicates in all cases. Results were tested for statistical significance using Kruskal–Wallis with Dunn’s multiple comparisons between culture condition within each cytokine, *P < 0.05 and **P < 0.001.

Previous studies of the bovine cytokine response have either focused on the systemic response or used a transcriptomics approach to whole tissues. Using a well-established model of experimental infection, we collected the MLN and HLN from infected animals after the development of patency. CD4 T-cells were isolated by magnetic bead selection and cultured for 72 h in the presence of anti-CD3 or LFH; thereafter, supernatants were collected for cytokine ELISA. In both the infected MLN (Figure 2A, right panel) and HLN (Figure 2B, right panel), there was no significant IFN-γ response either to CD3 or LFH; only CD3 stimulation of the MLN CD4 T-cells produced some detectable cytokine. This is in contrast to the findings in naïve tissues, where T-cells from both the MLN and HLN produced significant IFN-γ after anti-CD3, but not LFH stimulation (Figures 2A,B, left panels).

Of the type-2 cytokines examined, IL-5 was the most abundant at this point with CD3 stimulation but not LFH in the MLN and both CD3 and LFH stimulation within the HLN. In contrast, the naïve tissues produced IL-5 in response to anti-CD3 but not LFH; while IL-13 was non-detectable in naïve HLN T-cells low but variable levels were present in naïve MLN T-cells (Figures 2A,B, left panels). IL-13 was not significantly elevated except in response to LFH stimulation in the HLN. Indeed, only IL-10 and TGF-β were readily detectable in both the MLN and HLN. IL-10 was significantly upregulated within both the MLN and HLN in response to both CD3 and LFH, and for these responses in the HLN parasite specific IL-10 was more readily produced in comparison to CD3. TGF-β was spontaneously produced in PBS cultures in levels comparable to those produced by LFH. CD3-induced TGF-β was significantly raised, in comparison to both PBS and LFH, within the MLN but not the HLN (Figure 2B). IL-4 was not detectable in response to either CD3 or LFH at either site (data not shown). Overall, this would suggest that, even in the presence of a current infection, the draining HLN presents a strong immunoregulatory pattern with minimal type-2 cytokines.

### CD4 T-Cells from Infected Animals Are Activated but Do Not Express Foxp3

**Figure 3:**
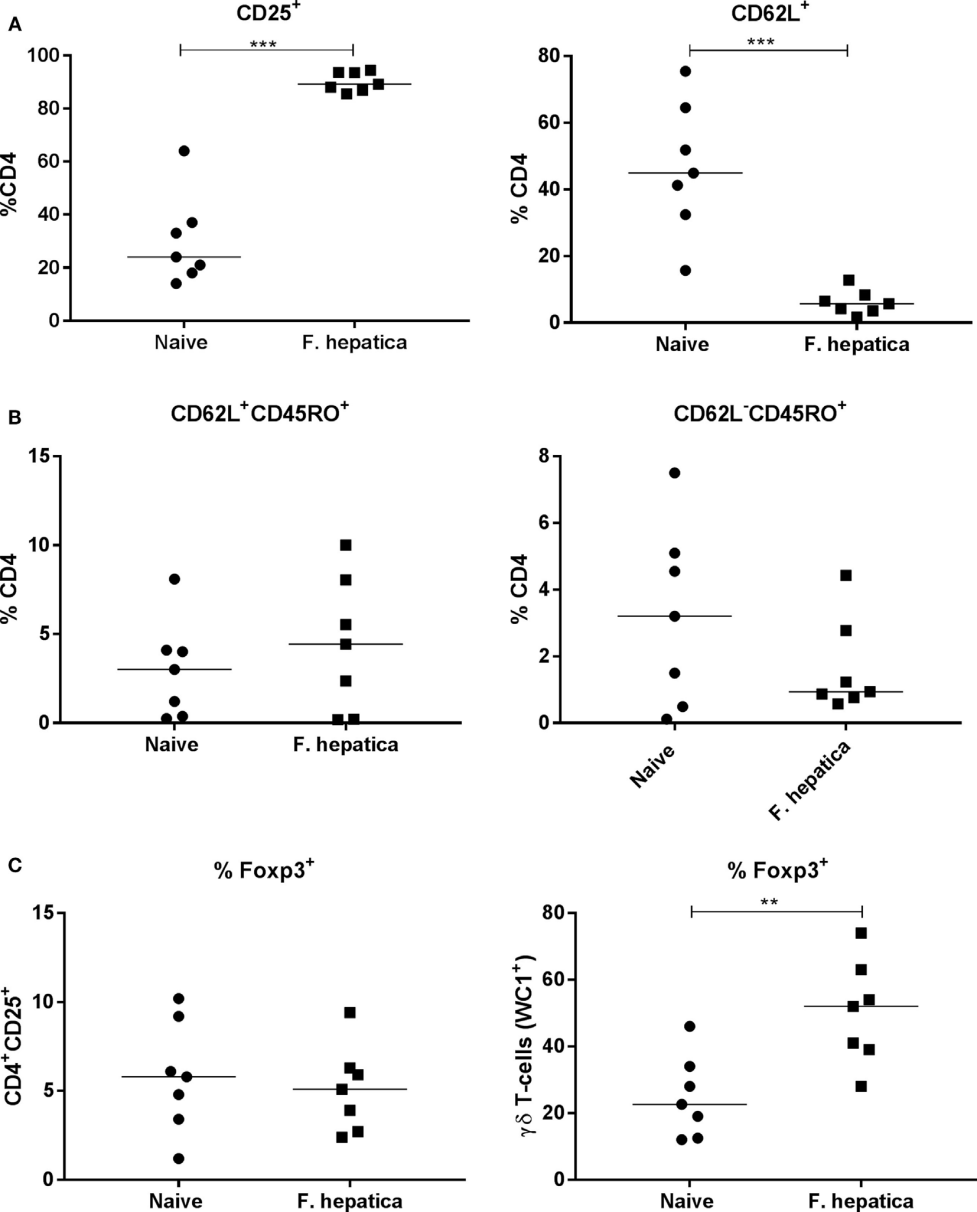
Activation, memory, and regulatory characteristics of the CD4 T-cell pool from infected lymph nodes. Single cell suspensions from the hepatic lymph node of week 13 *Fasciola hepatica-*infected animals (squares) or naïve controls (circles) were stained for CD4, CD25, CD62L, WC1, CD45RO, or intracellularly for Foxp3. **(A)** Shows an increase in the percentage of CD4^+^CD25^+^ cells, left, and a concomitant decrease in CD4^+^CD62L^+^ double-positive cells, right, indicating the presence of an activated CD4 population. **(B)** Staining for central memory T-cells (left) and effector memory T-cells (right) revealed that there was no significant change in the proportion of either of these populations although a noted wide variation was present in both naïve and infected animals. **(C)** Expression of the regulatory marker, Foxp3, was not changed in the CD4^+^CD25^+^ population when compared in naive and infected animals (left graph), but was found to be significantly upregulated in the γδ (WC1^+^) population between the two groups (right graph). Each point represents an individual animal. Significant differences between populations are indicated on the graphs determined using a Mann–Whitney *U-*test; **P < 0.01, ***P < 0.001.

We next examined CD4 T-cells from the HLN of infected animals, overall, there was a significant increase in the total number of cells from infected animals (95.2 ± 26.5 × 10^6^ cells; *P* < 0.05) compared with naïve controls (52.9 ± 20.4 × 10^6^ cells). The percentage of CD4 cells expressing CD25, as a marker of activation was 90 vs 30% within the controls (Figure 3A, left panel), in parallel, we saw a decrease in the percentage of naïve CD62L^+^ CD4 T-cells, an average of 44% in controls vs 6% from infected animals (Figure 3A, right panel).

To determine if the CD4 pool in the lymph node of infected animals was committed to a memory phenotype, we examined the CD4^+^CD62L^+^ subpopulation for CD45RO expression. There was no difference in CD45RO expression between infected and naïve animals (Figure 3B, left panel), 5.4 ± 1.8 and 4 ± 1% of the CD4^+^CD62L^+^ cells, respectively, suggesting no change in the commitment of T-cells to the central memory phenotype. Similarly, gating on the CD62L^−^ pool demonstrated that 3.2 ± 1% of naïve CD4 T-cells were also CD45RO^+^ while the corresponding population in infected animals was 1.7 ± 0.5%; demonstrating the lack of an memory cell expansion (Figure 3B, right panel). We stained the CD4^+^CD25^+^ T-cell population for Foxp3, we found only 5.8 ± 1.2% of infected animals, and 5.1 ± 0.9% of naïve animals, harbored traditional Foxp3 Tregs (Figure 3C, left panel). Indeed, we found that almost 50% (±6%) of γδ (WC1^+^) T-cells were Foxp3^+^ in infected animals compared with approximately half that proportion in naïve animals (Figure 3C, right panel). Thus, it would appear that the lack of proliferation and cytokine production in infected CD4 T-cells is not due to the presence of Foxp3^+^ CD4 T-cells.

### HLN CD4 T-Cells Appear Anergic

**Figure 4:**
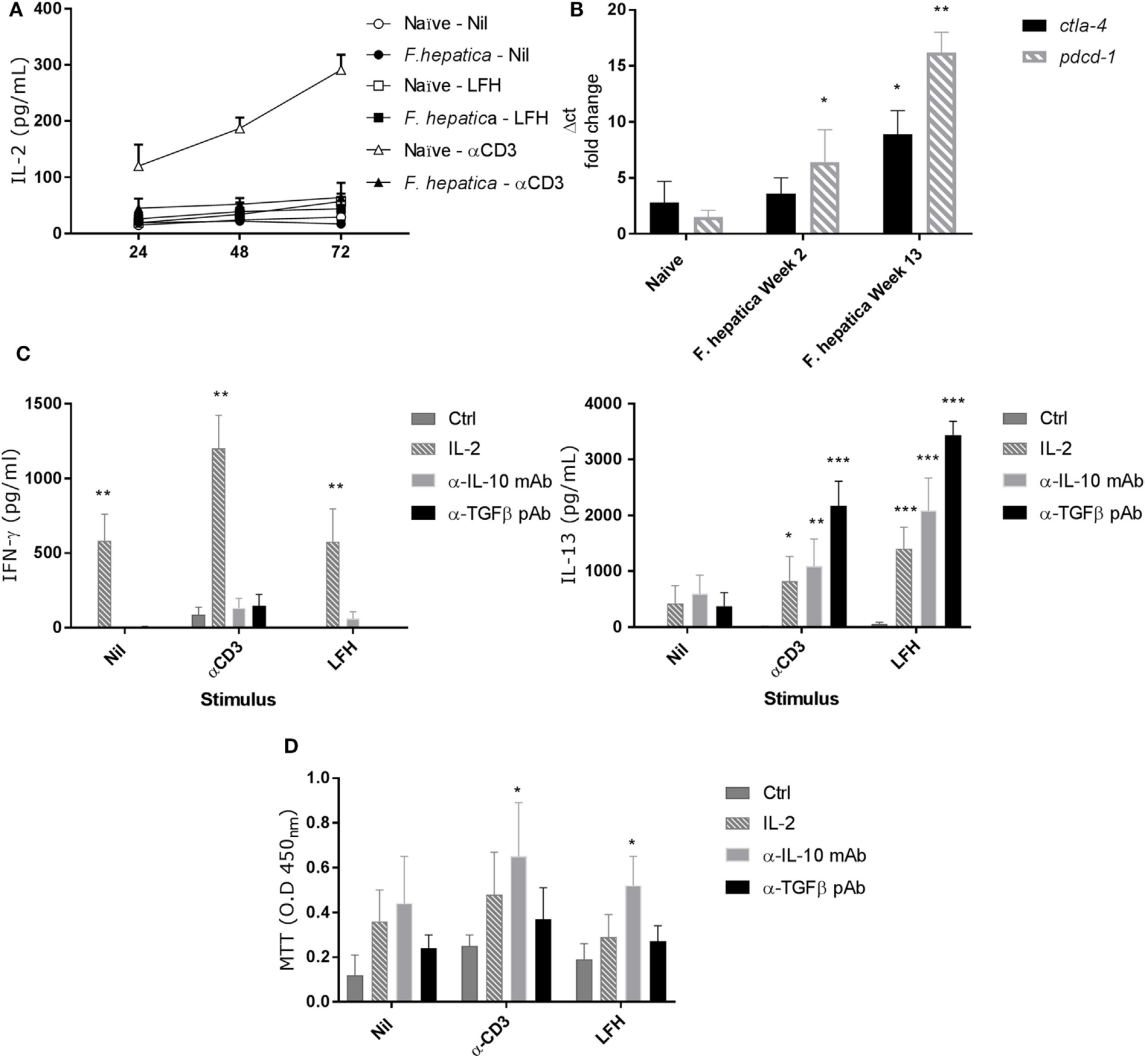
Failure to produce IL-2 and markers of anergy are coincident with non-responsive CD4 T-cells. **(A)** CD4 T-cells were isolated from the hepatic lymph node (HLN) of naïve or infected animals and cultured with α-CD3, LFH, or PBS (nil) for 72 h. Supernatants were recovered at every 24 h and tested for IL-2. There is a clear failure of CD4 T-cells from the HLNs of infected animals to secrete IL-2. However, naïve CD4 T-cells readily secrete IL-2 over time with a near linear accumulation of cytokine. **(B)** mRNA from CD4 T-cells was isolated, cDNA prepared, and qPCR carried out. There is a clear increase in *pdcd1* expression from week 2 onward when compared to naïve animals. *Ctla4* expression is significantly upregulated at week 13 but not week 2. **(C)** The effect of exogenous IL-2, or neutralizing IL-10 or TGF-β antibody on the expression of IFN-γ (left) and IL-13 (right) in infected CD4 T-cells from HLN was examined. There was a clear impact of IL-2 on IFN-γ, and IL-13 production was restored in the presence of all three treatments but to varying degrees. Ctrl represents cells treated with isotype Ab only at equal concentrations to neutralizing Ab. **(D)** CD4 T-cell proliferative responses in the HLN were assessed using the MTT dye assay after stimulation in the presence of treatments as in **(C)**. LFH and α-CD3 responsiveness was restored in the presence of α-IL-10 when compared within stimulation to non-treated controls. Bars represent the mean of seven animals ±SEM. Significant differences were determined using Friedman test with a *post hoc* comparison using a Wilcoxon ranked-sign test **(A,C,D)** and Kruskal–Wallis with Dunn’s multiple comparisons **(B)** where *P < 0.05, **P < 0.01, ***P < 0.0001.

IL-2 is a limiting factor in the responsiveness of lymphocytes, to determine if IL-2 played a role in the observed pattern of responses, we repeated the above CD4^+^ T-cell isolation and culture in the presence of LFH or α-CD3 stimulation. Cultures were sampled over a 72 h period, and Figure 4A shows that neither CD3 nor LFH drive IL-2 secretion but that naïve animals respond to CD3 stimulation with a steady accumulation of IL-2. Next, we conducted qPCR to determine if markers of anergy, *ctla4*, and *pdcd1* were upregulated. Both *ctla4* and *pdcd1* are upregulated in CD4 T-cells derived from animals 13 weeks of postinfection compared to those sampled at 2 weeks of postinfection or naïve controls (Figure 4B).

To test if the anergy could be reversed we repeated the above in the presence of IL-2, α-IL-10 (clone CC320), or α-TGF-β (AB-100-NA). While IFN-γ secretion was restored in response to LFH and CD3 with exogenous IL-2, neither α-IL-10 nor α-TGF-β had a significant effect on IFN-γ (Figure 4C). Conversely, IL-13 production was restored, compared to controls, in the presence of all three treatments during LFH and CD3 stimulation. Here, a graded effect was evident with an increasing capacity of IL-2, α-IL-10, and α-TGF-β treatments in restoring IL-13 production (Figure 4C). To determine if the mechanisms controlling cytokine production and proliferation were linked, we examined antigen-specific proliferation, by MTT assay, in the presence of the IL-2, α-IL-10, and α-TGF-β (Figure 4D). There was a high degree of variation in the responses of animals to these treatments. α-IL-10 treatment succeeded in significantly restoring the capacity of cells to respond to CD3 stimulation; while IL-2 or α-TGF-β had some effect this was not significant. Similarly, when stimulated with LFH, there was a small, but significant increase, in proliferation with α-IL-10 while neither IL-2 nor α-TGF-β had any effect on proliferation.

### Anergic T-Cells Suppress Autologous Antigen-Specific Responses

**Figure 5:**
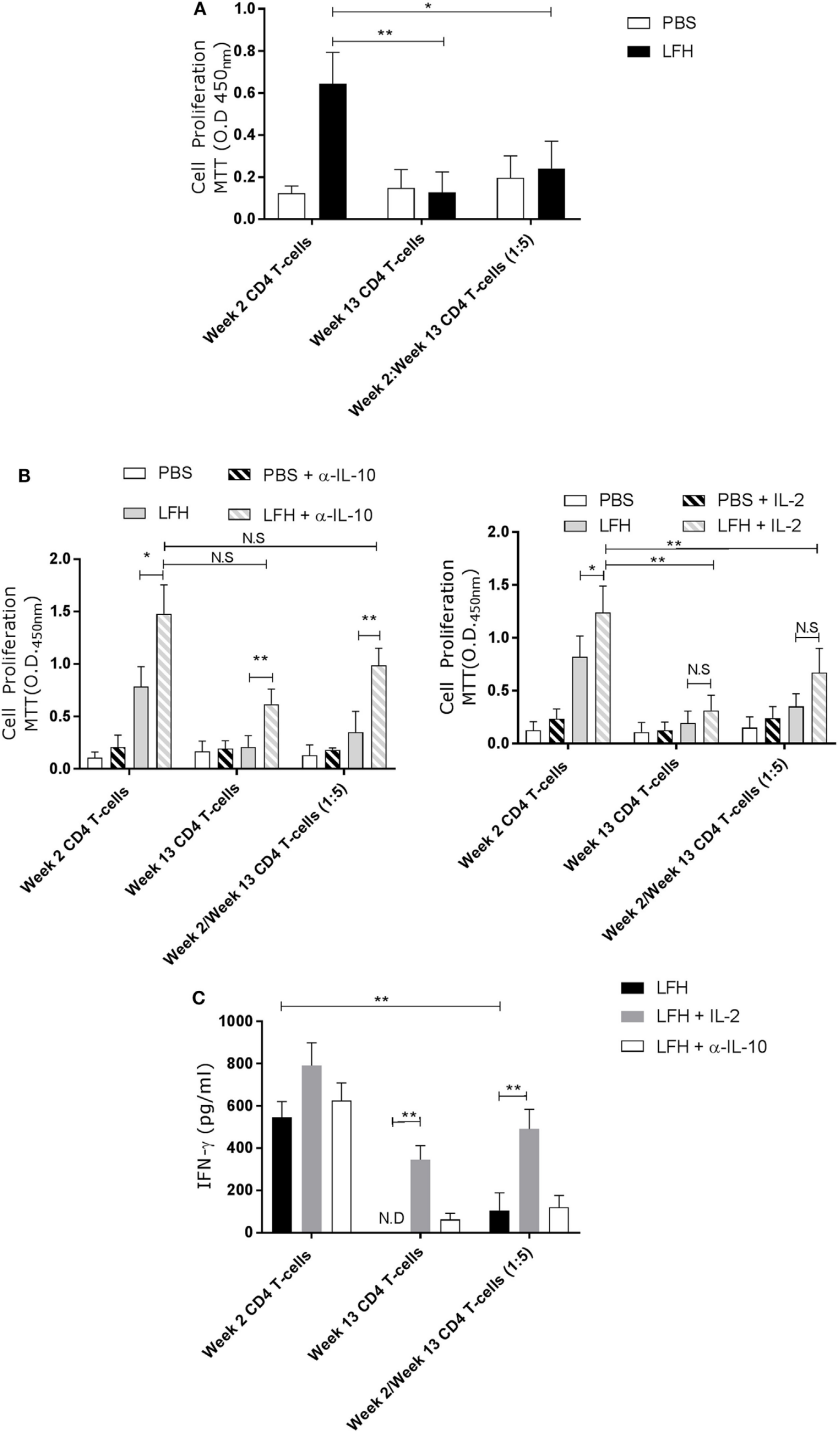
Anergic CD4 T-cells are suppressive toward autologous responsive counterparts. CD4 T-cells taken from animals’ 2 weeks of postinfection were cocultured with autologous CD4 T-cells isolated from the hepatic lymph node at postmortem. **(A)** Proliferation in response to LFH is still detectable in week 2 CD4 T-cells, as per previous findings; however, when anergic week 13 CD4 cells are added at a ratio of five cells per 1 week 2 responder cells, we see a strong suppression of proliferation. **(B)** A similar coculture was established and the effects of α-IL-10 (left graph) and exogenous IL-2 (right graph) was determined. In the presence of α-IL-10, there was significant reversal of the negative effects of anergic CD4 cells on their responsive counterparts; however, there was no positive effect of exogenous IL-2. **(C)** IFN-γ levels in LFH-stimulated cells untreated (isotype control) or treated with IL-2 and α-IL-10 were determined in the coculture system. In the presence of week 13 anergic CD4 cells, the IFN-γ response, from responsive week 2 cells, was suppressed while IL-2, but not α-IL-10 treatment, could abrogate the effect of week 13 CD4 T-cells on the responsive population. Bars represent the mean of seven animals’ ±SEM. Data in **(A)** and **(B,C)** were tested by Kruskal–Wallis with Dunn’s multiple comparisons and Friedman test with a *post hoc* comparison using a Wilcoxon ranked-sign test, respectively. ND, not detected; NS, not significant, *P < 0.05 and **P < 0.01.

The presence of these anergic T-cells may impact upon host responses to reinfection, or upon future vaccination. To test if the anergic T-cells could impact on normally responsive T-cells, we isolated CD4 T-cells from circulation at week 2 postinfection, when *F. hepatica-*infected animals are normally responsive, and cocultured these with anergic cells in the presence of LFH. While week 2 CD4 T-cells could respond normally, in the presence of week 13 cells, this response was abrogated (Figure 5A).

Addition of α-IL-10 resulted in an enhanced response in week 2 cells in response to LFH, suggesting that there was inhibition of low-level paracrine IL-10, and an increase in the week 13 only cells LFH-specific response (Figure 5B), consistent with our findings above. When α-IL-10 was added to the coculture, there was a reversal of the inhibitory effect of week 13 CD4 T-cells, and the IL-10 they produce, on week 2 CD T-cells. Neutralization of IL-10 in the cocultures of these cells resulted in a threefold increase in proliferation. Figure 5B demonstrating that pre-existing anergic T-cell can be a major contributor of IL-10, altering the response of freshly responding non-anergic T-cells. This same trend is visible in terms of IL-2 production and utilization. Addition of exogenous IL-2 in the same system had effects on week 2 only CD4 cells while no effect was apparent on week 13 CD4 T-cells. Figure 5B in our coculture system, the effect of IL-2 addition was weaker compared with IL-10 neutralization as a small but not significant effect was seen on LFH-specific proliferation (Figure 5C); suggesting that proliferative hyporesponsiveness in this setting is mediated by IL-10. To test if the dissociation seen in the actions of IL-2 and IL-10 extended to cytokine production, we examined the secretion of IFN-γ. Figure 5C clearly shows that IL-2 has a more profound effect on IFN-γ secretion in cocultured week 2 and week 13 cells when compared with α-IL-10 treatment; demonstrating that the cell-intrinsic effects we found in chronically infected animals can be influential on normally responsive week 2 cells.

## Discussion

We confirm a consistent pattern in chronic *fascioliasis* whereby Th2 cytokine responses are diminished by week 13 postinfection despite parasites residing in the liver. Broadly, this aligns with systemic PBMC responses at comparable stages of disease but diverges as there is an uncoupling of the regulatory mechanisms controlling Th2 and Th1 cytokines and cellular proliferation as they have been previously described (8, 18). Indeed, it would suggest that there are Th1 and Th2 intrinsic mechanisms controlling cytokine production on a per cell basis; supported by our finding that the CD4 compartment does not demonstrate expansion of a Foxp3 population. This aligns with findings from other models of *F. hepatica* and other veterinary parasite models. Escamilla et al. (19) have shown in goats and sheep that there is no expansion of CD4^+^Foxp3^+^ cells by 9 days of infection but, by 15–19 weeks of postinfection, an infiltrate of these cells surrounds the enlarged bile ducts of livers in sheep and goats. Interestingly, when the HLN from the same study was examined, there was little evidence of expansion of the CD4 Foxp3 population with no significant changes in sheep with only goats showing a ~6% increase over baseline during the acute infection stages localized to the cortex. The tendency for sheep and goats to harbor expanded CD4^+^Foxp3^+^ cells agrees with previous studies demonstrating that *Psoroptes ovis* causes an average of a 7% increase in recruitment of CD4^+^Foxp3^+^ cells to the skin (20). *Teladorsagia circumscintia* studies have demonstrated an expansion in the overall Foxp3 population in the intestine of sheep, but not specifically within the CD4 population (21). Prior studies demonstrated that the γδ (WC1) T-cell population maybe the major bovine regulatory population (22, 23). Recently, it has been shown that these cells can suppress both antigen-specific and non-specific responses within the context of foot-mouth-disease vaccination [23]. The effects of WC1^+^ γδ T-cells was shown to be dependent on IL-10 and TGF-β during these studies but independent of Foxp3; however, we did not characterize the effects of the WC1^+^ FoxP3^+^ population in this study. A definitive study of the function of Foxp3, in either CD4 or γδ T-cells, to demonstrate their specific actions within veterinary infectious disease remains to be completed. Tools are emerging that will allow an *in vivo* approach to the manipulation of CD4^+^Foxp3^+^ in some veterinary species already (24).

Previous studies of murine *F. hepatica* infection have shown similar effects to those demonstrated here. Aldridge and O’Neill (7) demonstrated a strong increase in markers of anergy, CTLA-4, and PD-1, within splenic CD4 T-cells; while little to no change was noted in the expression of FoxP3. Walsh et al. (25) studied the MLN and showed that there was no apparent role for Foxp3^+^ cells in the control of *F. hepatica*-specific responses. Our findings are more in keeping with these two latter studies than those using sheep or goats. We have demonstrated that an activated but anergic CD4 T-cell population controls the expression of IFN-γ and IL-13 by the production of IL-10, TGF-β, and to a lesser extent through the control of IL-2 activity; these same mechanisms of suppression were active in terms of cellular proliferation at the site of final parasite residence where there is a constant source of antigen. One of the key triggers for induction of anergy is a source of antigen in the absence of costimulation or the presence of ligands such as CTLA-4 (26).

Establishment of this phenotype within the HLN is problematic and may explain the failure of animals to develop resistance to reinfection. We demonstrated that anergic HLN T-cells can suppress autologous T-cells recovered from animals during the responsive period. In coculture, the addition of week 13 T-cells clearly and negatively impacted both proliferation and IFN-γ release. Anergic T-cells, which persist and overlap with a new incoming wave of parasites have the potential to skew the response of responsive T-cells *via* IL-10 production and altered IL-2 utilization. The uncoupling of the cytokine and proliferation control mechanisms is a novel finding given our previous findings (8); however, given that our study here is focused on lymphoid tissue sites and not circulating PBMCs, this is not unsurprising as secreting anti-inflammatory mediators is more likely effective in a systemic setting compared with controlling cytokine production/utilization at a single cell level. Recent studies have identified a PD-L1-expressing CD4 T-cell population as also being capable of suppressing IFN-γ in viral infection and blocking PD-L1 *in vivo* resulted in reductions in viral load (27). However, this was at a systemic level and our study here identifies a lymph node residing CD4 pool that differs to previous systemic circulating pools in terms of regulating mechanisms. Additionally, the uncoupling of the cytokine and proliferating control mechanisms would suggest that lymphoid tissue sites may influence the types of regulatory cells (or their mechanism) that arise differently to those that circulate or form in circulation.

Studies of murine schistosomiasis demonstrated that resistance to reinfection after a drug-abbreviated infection was improved in the context of anti-IL-10 treatment (28). Blockade of IL-10 resulted in a global increase in the antigen-specific Th1/Th2/Th17 responses, an attractive proposition given the prevailing thoughts that ruminants are protected from *F. hepatica* only through a combined Th1/Th2 response (29). While in human schistosomiasis, there was significant effect of increases in posttreatment Th2/Th17 cytokine responses and protection against reinfection (30). Whether these effects can be extended to fascioliasis remains to be examined. Moreover, to fully appreciate the role of anergy in the response to fascioliasis, we must consider all findings in a breed-specific context and the influence of MHC haplotype will guide the development of anergy given its reliance on antigen exposure and the variation within MHC complexes that has long been recognized in different cattle breeds (31). However, our data here were obtained from Holstein–Friesian cattle, which are the most common breed within the UK in terms of dairy production (32) and so are highly representative of the dairy population.

In conclusion, we have shown the presence of an antigen-specific CD4 T-cell population, which appears to be hyporesponsive from the initial stages of chronic infection. These cells upregulate CTLA-4 and PD-1, but not Foxp3, and are both suppressive in a cell-intrinsic manner and in the context of autologous-responsive cells. This is mediated by IL-10 secretion and control of IL-2 production both of which can be reversed *ex vivo* to restore both cytokine production and proliferation. Importantly, these mechanisms are uncoupled in that IL-2 and IL-10 have distinct actions, which appear not to overlap. It remains to be demonstrated how long these cells persist during infection and posttreatment in the context of Fascioliasis, and whether they have a functional role in mediating the lack of resistance to reinfection despite repeated drug-abbreviated infections.

## Ethics Statement

Experiments were conducted under Home Office License PPL 30/3239 in accordance with Animal Scientific Procedures Act 1986 and ethical approval for the study was provided by the University of Nottingham SVMS and ADAS UK Animal Welfare and Ethics Review Committee. Animals involved in experiments had their suffering minimized and their welfare was maintained as outlined in the guidance provided by EU Directive 2010/63/EU.

## Author Contributions

DS and RF performed experiments; RF and KG oversaw study design; DS, KG, and RF contributed to data analysis; DS, RF, and KG prepared final draft.

## Conflict of Interest Statement

The authors declare that the research was conducted in the absence of any commercial or financial relationships that could be construed as a potential conflict of interest. The reviewer, MM, and handling editor declared their shared affiliation, and the handling editor states that the process nevertheless met the standards of a fair and objective review.
